# Supplementary material for: Movement errors during skilled motor performance engage distinct prediction error mechanisms
Source: Commun Biol. 2020 Dec 11;3:763. doi: 10.1038/s42003-020-01465-4 (PMC7732826; doi:10.1038/s42003-020-01465-4)
Supplement: Supplementary file 1 — Supplementary Information [file 42003_2020_1465_MOESM1_ESM.pdf]

Movement Errors During Skilled Motor Performance Engage Distinct Prediction Error Mechanisms

Ella Gabitov[1,2,1], Ovidiu Lungu[1,2], Geneviève Albouy[3], Julien Doyon[1,2,*]

[1]McConnell Brain Imaging Center, Montreal Neurological Institute, Montreal, Quebec H3A 2B4, Canada

[2]Department of Neurology and Neurosurgery, Montreal Neurological Institute, McGill University, Montreal, Quebec, H3A 2B4, Canada

[3]Movement Control and Neuroplasticity Research Group, Department of Movement Sciences, KU Leuven, Leuven 3000, Belgium

[1] Correspondence:
gabitovella@gmail.com or julien.doyon@mcgill.ca

SUPPLEMENTARY NOTE 1

Performance during task (re)initiation. We instructed participants to restart the sequence from the first key and to continue with the task as smoothly as possible if they noticed that they made an error, rather than trying to correct it midstream. Once the predetermined order of keypresses is violated, trying to figure out the last correct keypress and the next key that should have been pressed may not be a trivial task. Our guidelines, however, did not require such an effort from participants; instead they were provided with a clear and easy approach to deal with errors. Could the post-error slowing simply reflect such performance reset? To explore this possibility, we conducted an additional analysis of behavioral data comparing post-error performance with successful initiation at the start of the blocks; performance during sequences position-matched to the first post-error sequence was also included in the analysis and was used as a control condition. For each individual, we matched the number of the first-block sequences considered in the analysis with the number of error periods by applying a random selection procedure from all correctly initiated blocks. In this way, the statistical power to detect changes was comparable across different sequence/trial types. The results of this analysis indicate that the post-error slowing is of a greater magnitude than slowing after rest, even though in the letter case there is a need for exogenous attention to the GO cue (Supplementary Fig. 2).

A neural signature of valence PES. There is evidence suggesting that the NAc may be involved in processing of salient events without any reward, feedback or motivational value [1–3] (for meta-analysis, see [4–7]) and may also promote action initiation [8]. Although these observations and the role of the NAc in signaling positive valence PES are not mutually exclusive, they complicate the interpretation of activity increases within this structure during unexpected positive outcomes. Negative effects within the NAc during unexpected negative outcomes, on the other hand, are incompatible with the salience and action initiation account – brain circuits involved in these processes should exhibit activity increases – and, therefore, constitute a more reliable neural signature for valence PES.

Mesolimbic and nigrostriatal dopaminergic pathways. The putamen in primates and its homolog within the dorsolateral striatum in rodents is known to integrate incoming action-related information from sensorimotor cortices [9,10]. In addition, similar to the NAc, this sensorimotor afferent receives projections from the midbrain dopaminergic system and, as such, may also be implicated in reward PES and outcome valuation [11]. However, whereas NAc is innervated by dopamine-producing cells in the ventral tegmental area, the sensorimotor striatum receives dopamine input from the substantia nigra [12], hence forming respectively the mesolimbic and nigrostriatal dopaminergic pathways [13,14]. Traditional theories ascribe distinct functions to these two pathways. While the mesolimbic pathway has been implicated in reward processing, the nigrostriatal pathway is believed to be crucially involved in motor control and action selection. Although, emerging evidence from animal studies

indicates that under certain conditions such dichotomy may be violated [15,16], our results suggest that during skilled motor behavior, the division of labor between the striatal targets of the mesolimbic and nigrostriatal pathways (i.e., NAc and putamen, respectively) is consistent with the classic view on their dissociative function.

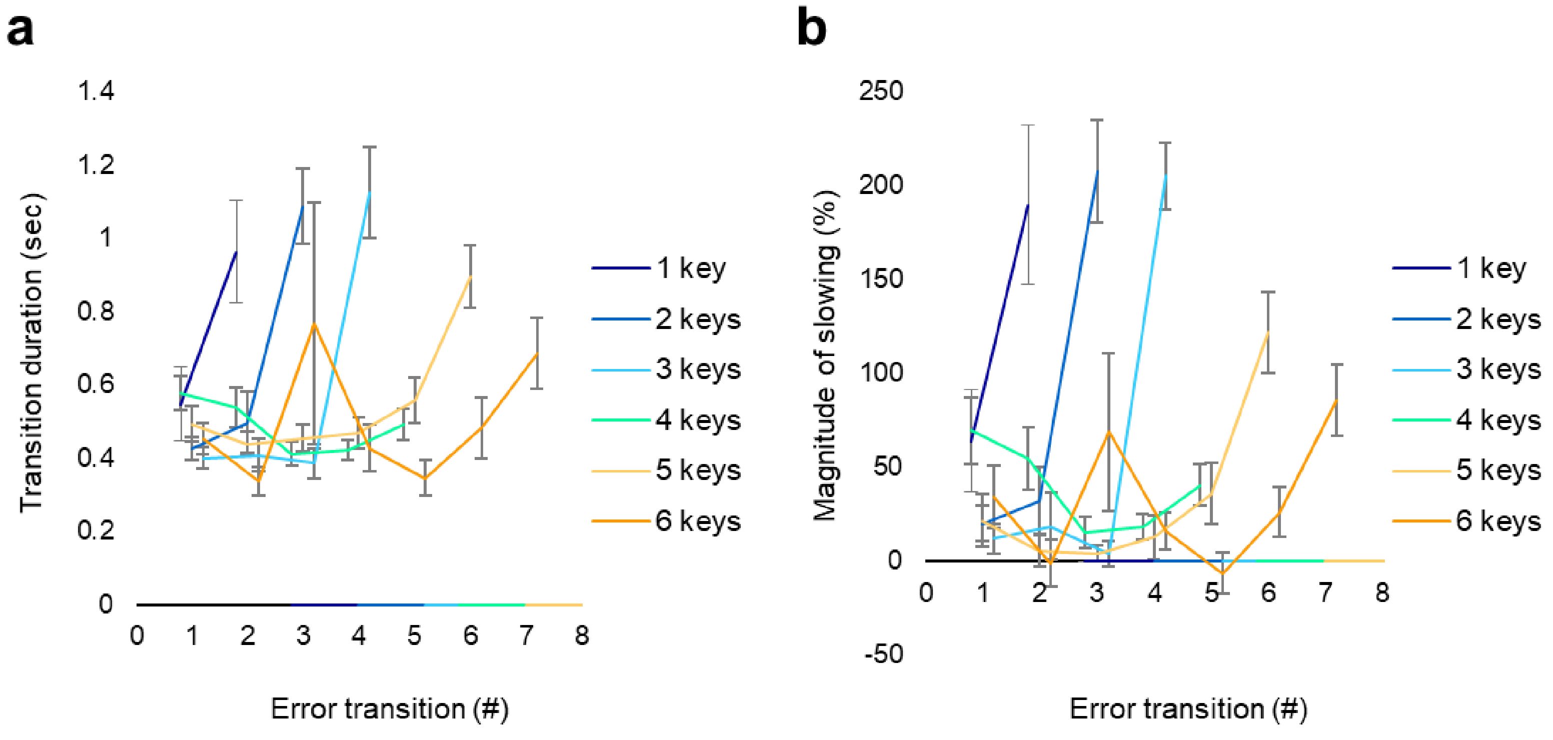

Supplementary Fig. 1 Changes in tapping speed during errors. a Mean time to complete a single transition (i.e., transition duration) and **b** the magnitude of slowing during errors with different lengths. Each line connects the mean values of a single transition during errors grouped by their length (from 1 to 6 keypresses). The first and the last values represent mean across transitions at the error onset and offset, respectively. Error bars represent standard error of the mean (SEM).

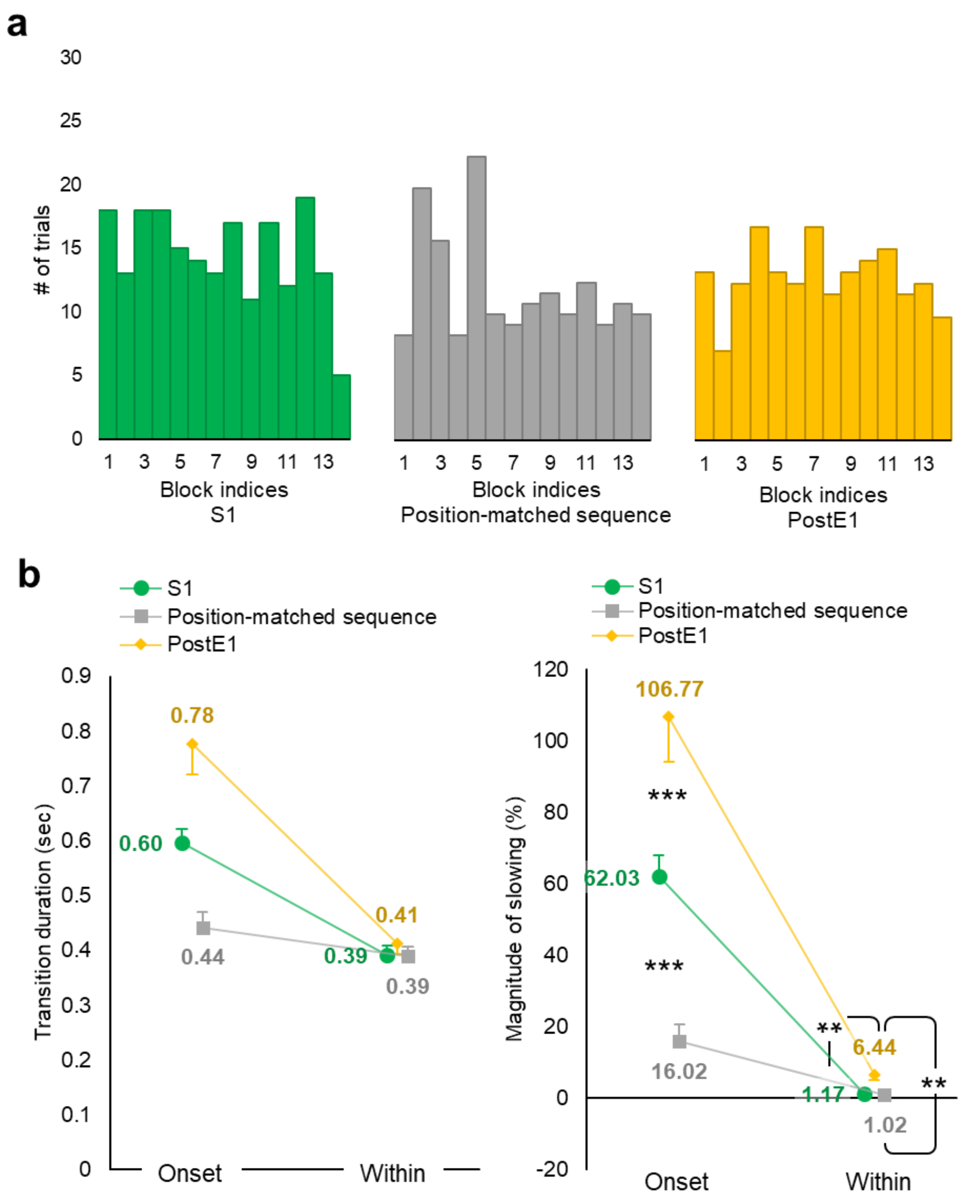

Supplementary Fig. 2 Performance during task (re)initiation. a Distribution of the trials (i.e., sequences) across blocks. The analysis of the distribution of trials of each type (i.e., first, position-matched and post-error sequences) across blocks shows no significant results (effect of *block*: $F(13, 624) = 1.532$, $p = 0.101$; *block* by *trial type* interaction: $F(13.55, 650.47) = 1.294$, $p = 0.208$), suggesting non-heterogeneous and equivalent trial distribution. **b** Mean duration and magnitude of slowing of transitions to trial onsets and within trials. Group means are shown for the first-block sequence (S1, green circles), the mid-block sequence position-matched to the first post-error sequence (position-matched sequence, gray squares), and the first post-error sequence (PostE1, yellow diamonds). For each subject, the number of the first-block sequences considered in the analysis was equal to the number of error periods (random selection from all correctly initiated blocks). In that way, the statistical power to detect changes was comparable across different trial types. Both, the onset and execution of the post-error sequence are significantly slower than those of the first and position-matched sequences ($|t(48)| > 2.595$, $p <= 0.013$). **/*** – significant differences at 0.01/0.001 level. Error bars represent standard error of the mean (SEM).

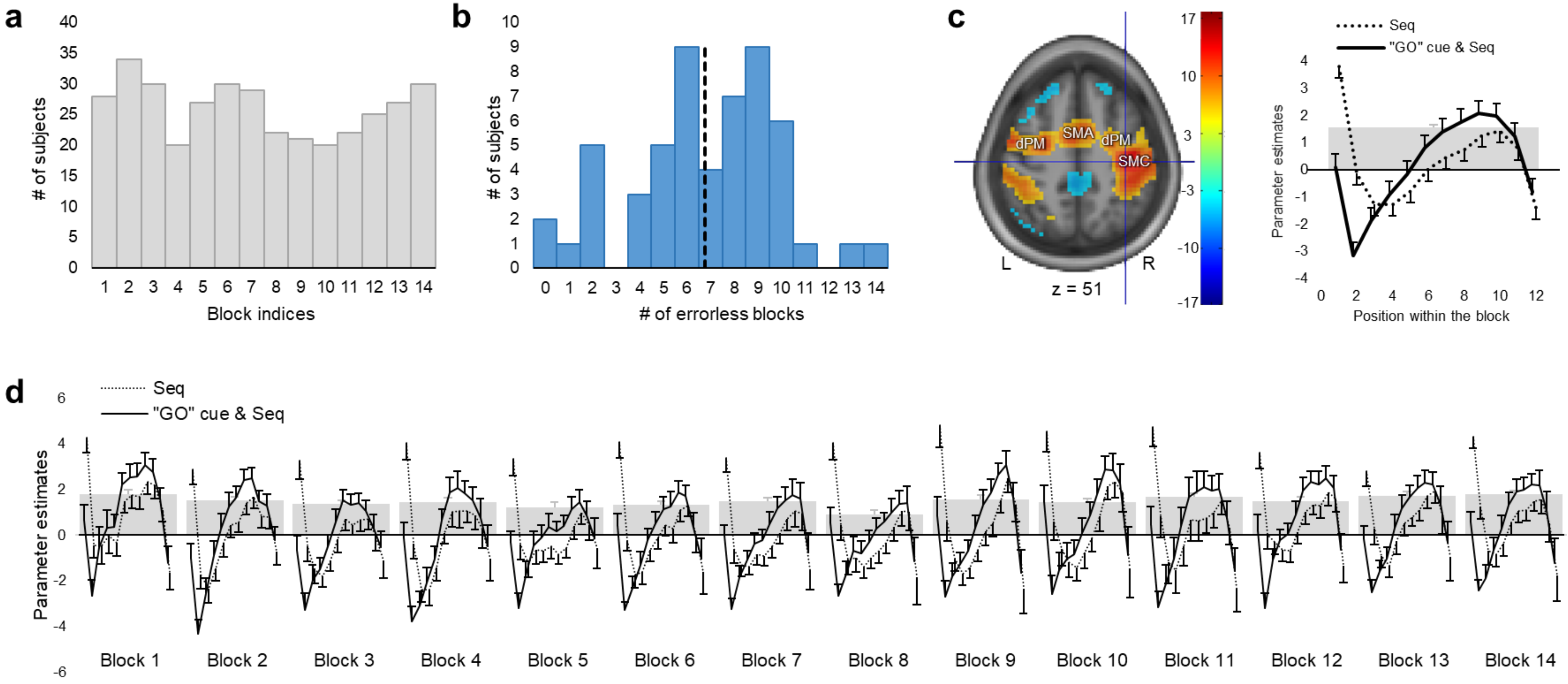

Supplementary Fig. 3 Within-block changes in activity during errorless blocks. a The number of participants with errorless performance for each performance block (1 – 14). On average, 26 individuals completed one block with no error (i.e., 12 correct trials/sequences) (mean = 26.07 participants, SEM = 1.19). **b** The distribution of the number of errorless blocks across participants. The vertical dashed line represents the group mean of the number of errorless blocks for the entire sample. After excluding two participants with no errorless blocks, the data of 52 individuals were used to access neural activity during errorless performance (mean = 7.02 errorless blocks, SEM = 0.40). **c** Task-related network and within-block changes in activity during errorless blocks. We used mixed block/event-related design modeling performance periods as epochs/blocks and trials/sequences as events. Activation map with the main effect of errorless blocks is displayed at $p < 0.05$, FWE-corrected at the peak level (performance blocks > rest) (Supplementary Table 1). Increased BOLD responses were observed within the characteristic motor network and were paralleled by decreased activity within the default-mode network. Parameter estimates were extracted for each trial within errorless blocks using sphere ROI centered at the local maxima within the hand area of the (right) primary sensorimotor cortex contralateral to the performing hand (r = 6 mm, xyz = 36, -21, 51) (dashed line). Note, that inclusion of performance onset (GO cue) as an additional event-related covariate in the model (solid line) contributed to faster activity restoration. Gray bar represents mean task-related activity averaged across errorless performance blocks (errorless blocks > rest). Data-points represent parameter estimates for each trial position within the block (1 – 12) averaged across performance blocks while controlling for the mean task-related activity. L and R – left and right hemisphere, respectively. SMC – sensorimotor cortex, SMA – supplementary motor area, dPM – dorsal premotor cortex. **d** Within-block changes in the BOLD signal, extracted from the contralateral (right) sensorimotor cortex (described in b), are plotted separately for each block. Gray bars represent group mean of task-related activity for each performance block. Dashed and solid lines connect between mean parameter estimates extracted from models without and with a regressor for the block onset (i.e., GO cue), respectively. Data-points represent group mean of parameter estimates for each trial within the block (1-12). Error bars represent standard error of the mean (SEM).

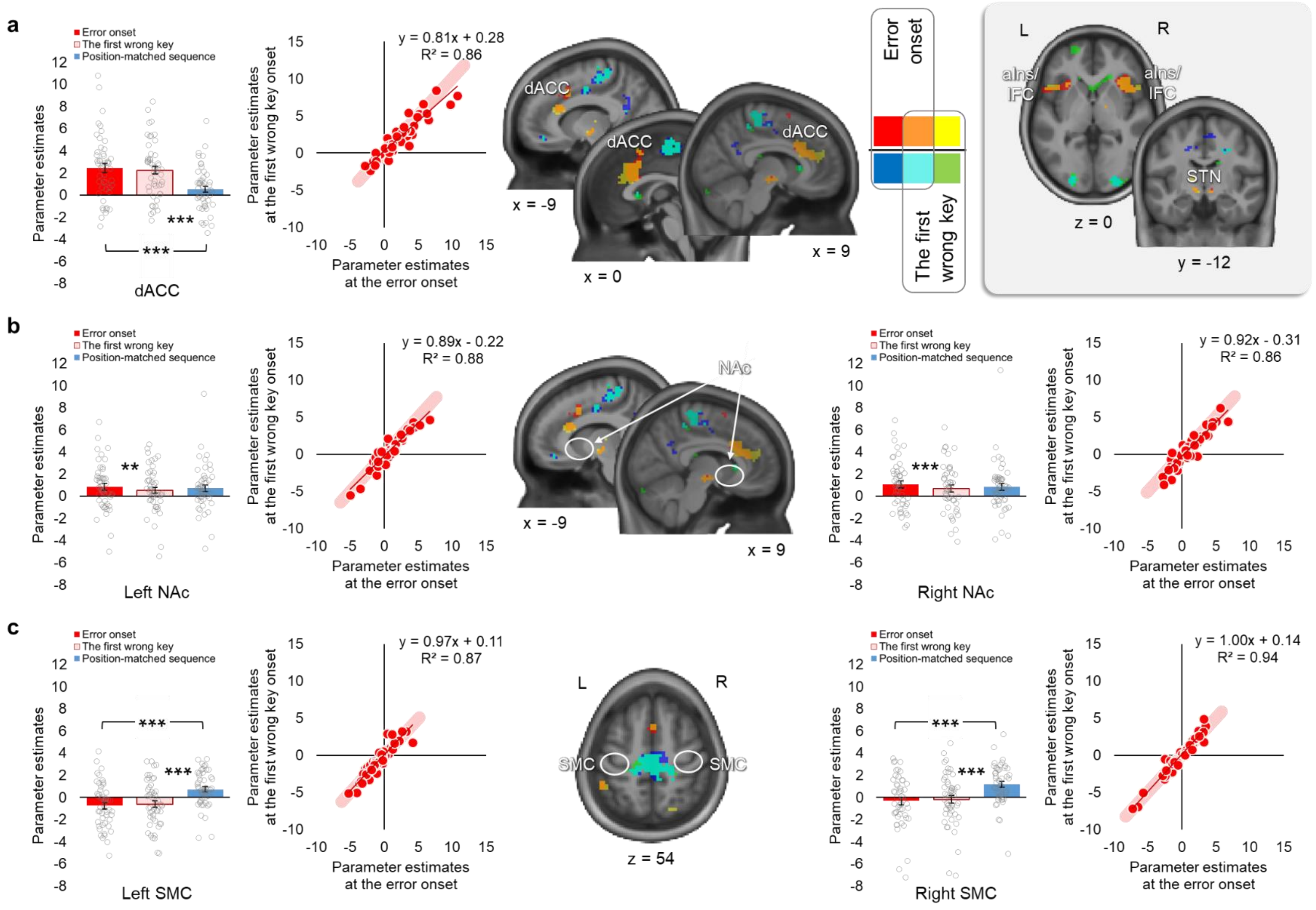

Supplementary Fig. 4 Main effect of the error onset and of the first wrong keypress. Parameter estimates extracted from regions of interest (ROIs) within **a** the dorsal anterior cingulate cortex, **b** the nucleus accumbens, and **c** primary sensorimotor cortices. The results of the whole-brain analyses, thresholded at $p < 0.001$, are shown as colored clusters: red/blue and yellow/green – areas with increased/decreased activity at the error onset and at the first wrong keypress, versus rest, respectively; orange/cyan – areas with significant activity increases/decreases in both conditions (i.e., overlap). Data-points on scatter plots represent parameter estimates extracted from sphered ROIs for each individual. The wide pink line marks equal values across conditions. Columns represent parameter estimates averaged across participants for each condition; mean parameter estimates at the onset of the position-matched sequence is also shown. L/R – left/right hemisphere. dACC – dorsal anterior cingulate cortex, aIns/IFC – anterior insula/inferior frontal cortex, STN – subthalamic nucleus, NAc – nucleus accumbens, SMC – sensorimotor cortex. **/*** – significant results at 0.01/0.001 level. Error bars represent standard error of the mean (SEM).

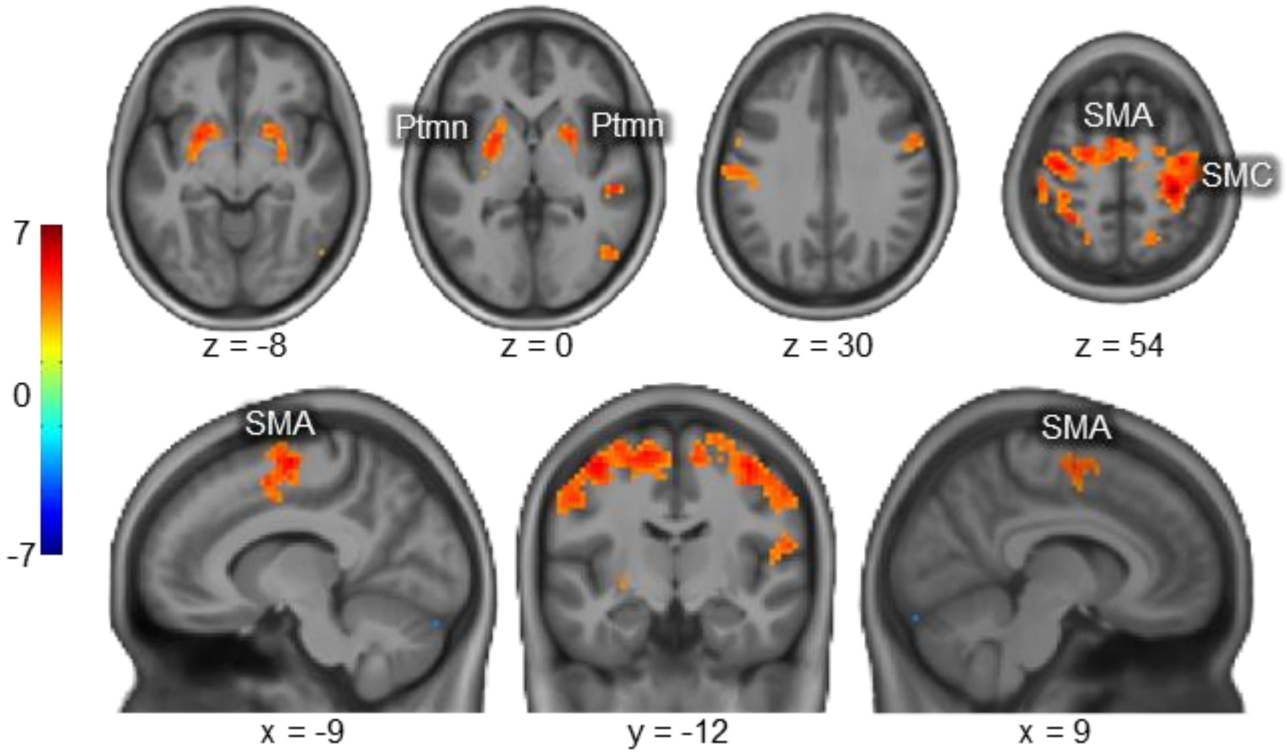

Supplementary Fig. 5 Main effect of position-matched sequences. Activation map with areas showing significant changes in BOLD signals during position-matched sequences versus rest, while controlling for the mean task-related activity using mixed block/event-related design, is displayed at $p < 0.001$. Increased BOLD responses were observed within the characteristic motor network including contralateral primary sensorimotor cortex, supplementary motor area and dorsal premotor and parietal cortices, bilaterally. At the subcortical level, significant positive effects were found within the bilateral putamen. Color bar represents *t* values. SMC – sensorimotor cortex, SMA – supplementary motor area, Ptmn – putamen.

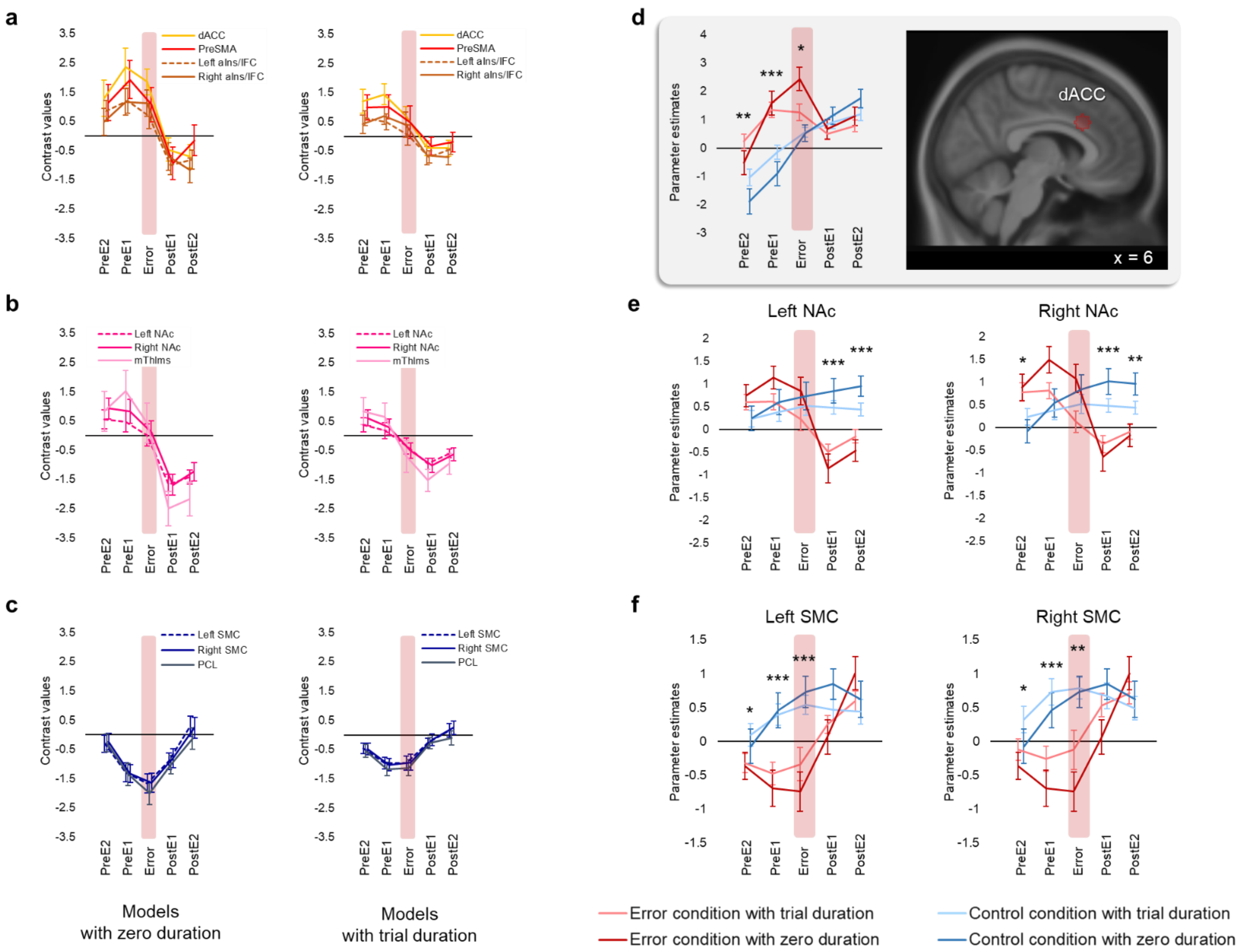

Supplementary Fig. 6 Temporal characteristics of changes in activity during error processing using models with zero and actual trial durations. Activity levels during periods with errors, estimated relative to the control error-free task condition (i.e., values along the x-axis), are plotted for areas with **a** increased activity during errors, **b** decreased activity during post-error trials, and **c** decreased activity during errors. Activity levels during periods with and without errors versus rest (error and control condition, respectively) are plotted for **d** the dorsal anterior cingulate cortex (dACC), **e** the left and right nucleus accumbens (NAc), and **f** left and right sensorimotor cortex (SMC. Black asterisks indicate significant differences in activity between corresponding trials during periods with and without errors modeled with trial duration (light red and light blue lines, respectively). The results derived from analyses using event-related design (i.e., zero trial durations) are reported in the main text. */**/*** – significant results at 0.05/0.01/0.001 level; in the latter case, the results are significant after Bonferroni correction for 25 ROIs included in the analysis ($p < 0.002$). Error bars represent standard error of the mean (SEM).

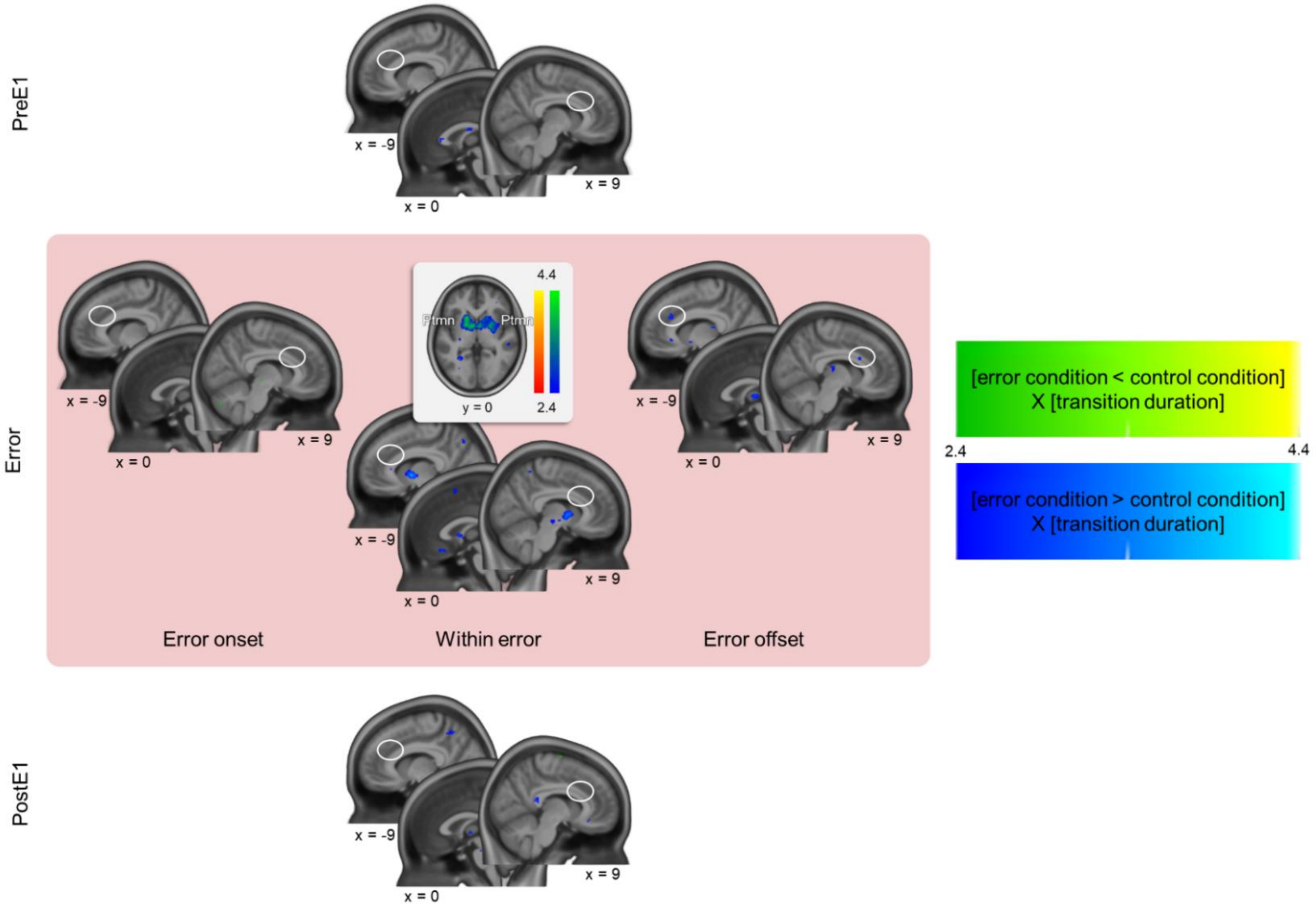

Supplementary Fig. 7 Changes in activity associated with durations of different trial phases. Areas in which increased activity levels are associated with longer transition durations during the correct trials immediately before and after the error (PreE1 and PostE1, respectively) and during the error itself. Only subjects with more than two errors (N = 38) were included in the analysis. Separate analyses were performed for error onset, within error transitions and error offset. The effects were estimated relative to the control condition. Trials and error phases of interest were modeled as events whereas transition durations were included as parametric modulators. The group maps showing differential changes in activity associated with transition durations during periods with and without errors (error and control condition, respectively) were initially thresholded at a very liberal level of 0.01; a *p*-value of 0.05 was used for cluster-level inferences. The color bars indicate *t*-values. No regions exhibit significantly lower activity levels during periods with errors than during periods without errors as a function of transition durations (green-yellow colors). No significant changes associated with transition durations were found within the dorsal parts of the anterior cingulate cortex (dACC, white circles). Significant [error condition > control condition] by transition duration interaction (blue-turquoise colors) is observed within the bilateral putamen (Ptmn; with local maxima at [-18, 6, 0] and [24, 0, 3]). The post-hoc analysis performed separately for each condition showed that this interaction is driven by increased activity associated with shorter transition durations during correct trials matched to errors (the white inserted panel). There was no significant effect associated with longer transition durations (red-yellow colors).

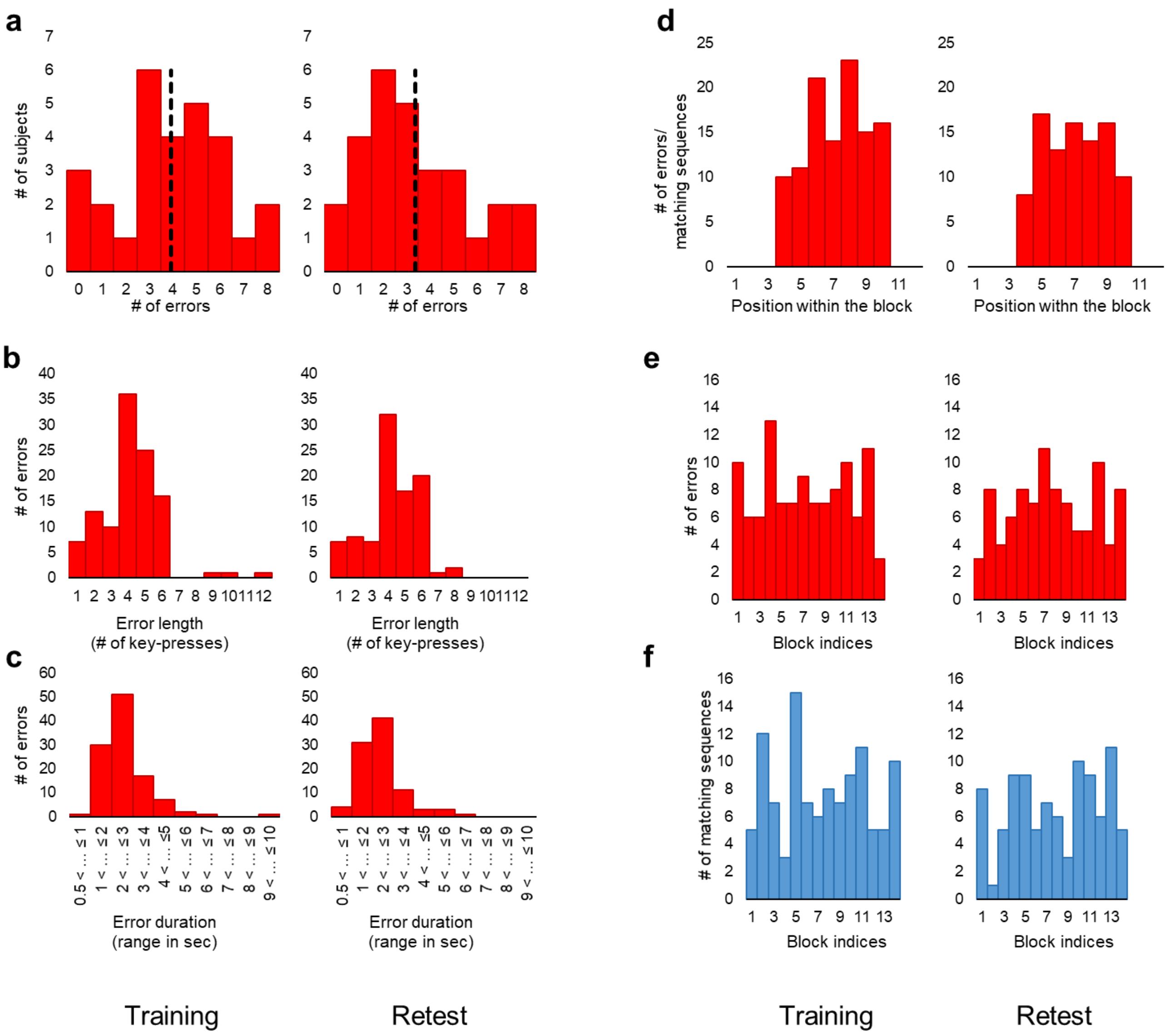

Supplementary Fig. 8 Sample summary for errors and position-matched sequences for the sub-group of participants who were trained and retested on the same motor sequence. a The distribution of number of errors across participants. The vertical dashed line represents the group mean of the number of errors. **b, c** The number of errors plotted against their length (i.e., the number of keypresses within the error) and duration (i.e., the total time spent on the error including transitions to its first and from its last key; intervals are given in seconds), respectively. **d** The distribution of errors/position-matched sequences within performance blocks. **e, f** The distribution of errors and position-matched sequences, respectively, across performance blocks.

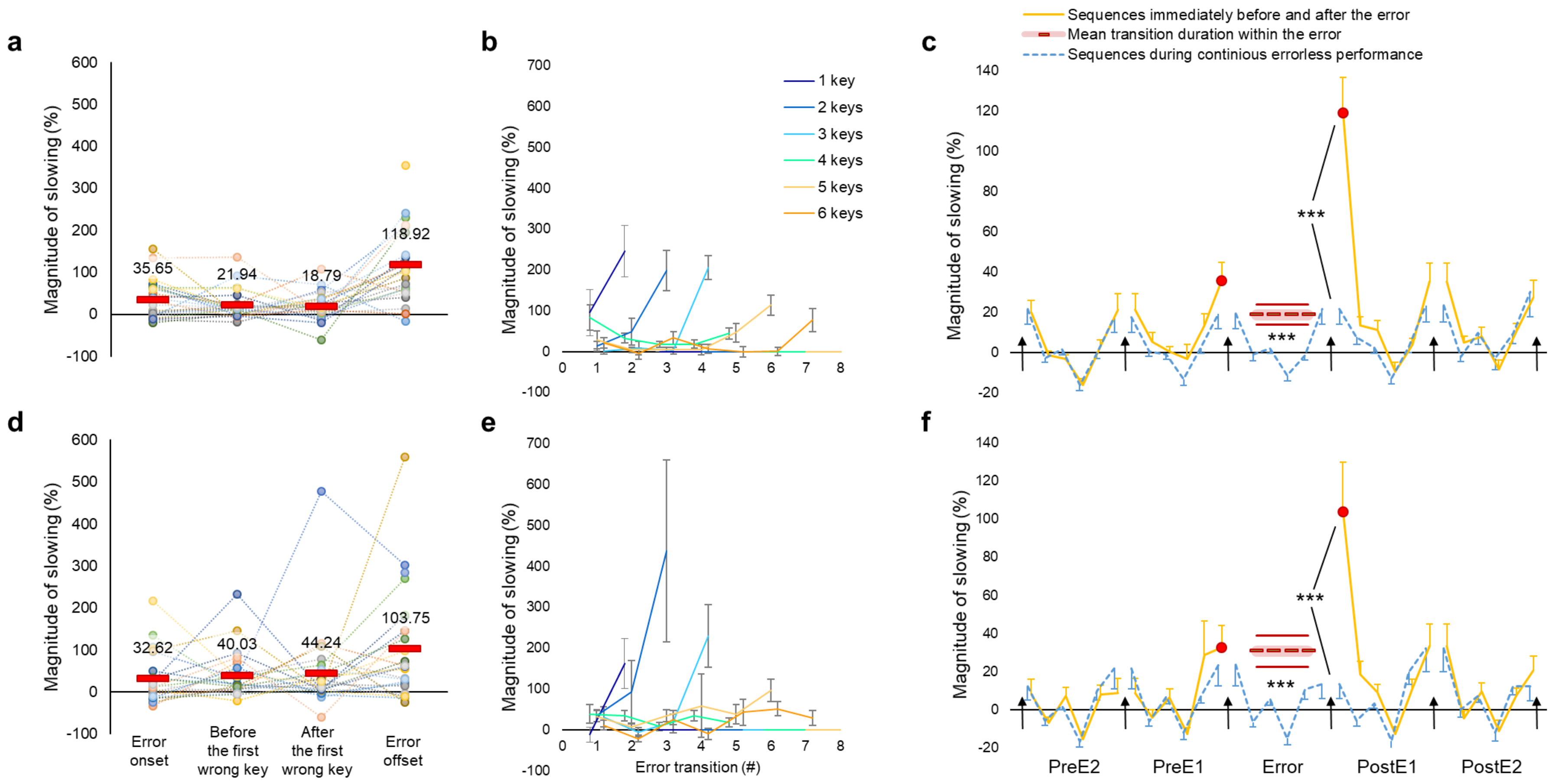

Supplementary Fig. 9 Behavioral results for the sub-group of participants who were trained and retested on the same motor sequence. a-c Training session. **d-f** Retest session. **a, d** The magnitude of slowing at the error onset, within the error (mean values for transitions before and after the first wrong keypress are shown separately), and at the error offset. Each data-point represents mean values calculated across errors separately for each individual. Red markers indicate the group mean. **b, e** The magnitude of slowing during errors with different lengths. Each line connects the mean values of a single transition during errors grouped by their length (from 1 to 6 keypresses). The first and the last values represent mean across transitions at the error onset and offset, respectively. **c, f** The magnitude of slowing immediately before, during and immediately after errors in comparison to the corresponding trials during position-matched periods of errorless performance. Orange lines connect mean values for transitions within and between two adjacent sequences immediately before and after the error. Blue dashed lines connect mean values for transitions within and between sequences during position-matched periods of errorless performance. Black arrows indicate transitions between sequences. Red circles represent mean values for transitions to the first and from the last error key (i.e., error onset and offset, respectively). Dashed and continuous red lines represent the mean magnitude of slowing and SEM. within the error, respectively. Zero values for the magnitude of slowing (the x-axis) represent the mean transition duration within all correct trials; these values were initially calculated separately for each block and each individual, and were used to estimate the magnitude of slowing on the block-by-block basis. PreE2 – penultimate sequence before the error, PreE1 – last sequence immediately before the error, PostE1 – first sequence immediately after the error and PostE2 – second sequence after the error. */***– significant results at 0.05/0.001 level. Error bars represent standard error of the mean (SEM).

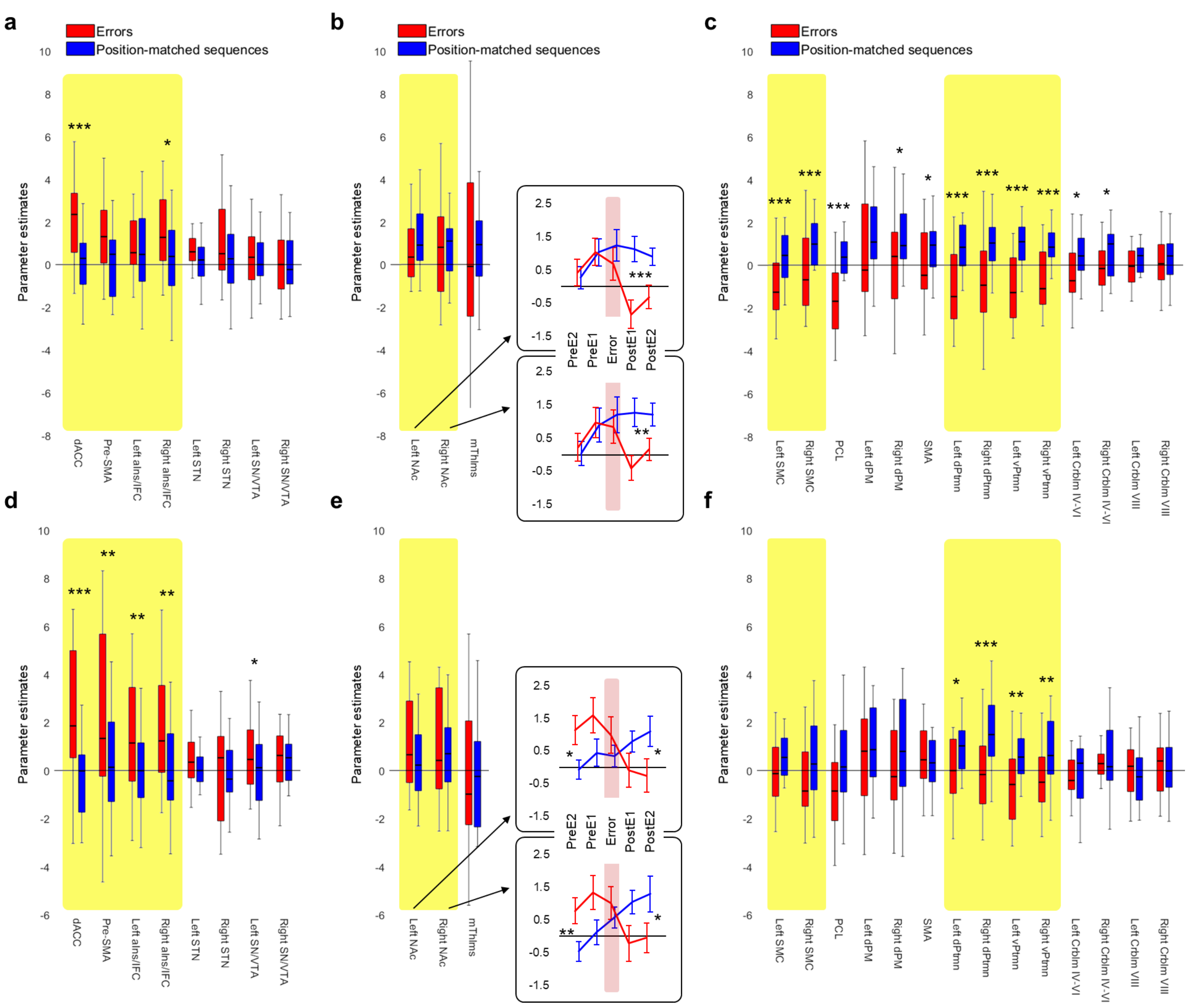

Supplementary Fig. 10 Activity levels during errors and position-matched sequences versus rest for the sub-group of participants who were trained and retested on the same motor sequence. a-c Training session. **d-f** Retest session. Parameter estimates at the onset of the errors (red) and position-matched sequences (blue) extracted for regions of interest (ROIs) with error-specific activity increases, post-error activity decreases and error-specific activity suppression. We used the same ROIs as in our main study (Table 3). Contrast values for ROIs highlighted in yellow are shown in Fig. 7. Black asterisks indicate significant differences between errors and position-matched sequences; red and blue asterisks indicate significant activity increases/decreases, versus rest, during errors and position-matched sequences, respectively. */**/*** – significant results at 0.05/0.01/0.001 level; in the latter case, the results are significant after Bonferroni correction for 25 ROIs included in the analysis ($p < 0.002$). Error bars represent standard error of the mean (SEM).

Supplementary Table 1. Main effect of blocks versus rest during errorless performance.

Area	MNI coordinates			Size		
	x	y	z	(# of voxels)	*t*	*p*
1. Increased task-related activity (errorless blocks > rest)						
Cluster 1				1957		< .001 FWE
Left cerebellum IV-V[22]	-18	-51	-24		16.42	
Left cerebellum VI	-30	-45	-33		12.19	
Right cerebellum VI[23]	24	-57	-27		15.07	
Left cerebellum VIII[24]	-18	-60	-51		14.65	
Right cerebellum VIII[25]	24	-57	-54		12.51	
Vermis IV	0	-48	-3		10.78	
Cluster 2				5101		< .001 FWE
Right precentral gyrus	39	-21	63		14.12	
Right precentral gyrus[13]	36	-21	51		13.99	
Right postcentral gyrus	39	-27	45		13.10	
Right frontal superior gyrus[16]	27	-6	60		10.23	
Left SMA[17]	-6	-3	57		13.92	
Left precentral gyrus	-45	-9	-54		11.88	
Left precentral gyrus[15]	-27	-9	57		11.86	
Left frontal superior gyrus	-30	-9	69		9.77	
Left postcentral gyrus	-51	-21	21		9.57	
Left inferior parietal gyrus	-51	-24	39		10.84	
1. Decreased task-related activity (errorless blocks < rest)						
Cluster 1				4530		< .001 FWE
Right middle occipital gyrus	36	-84	27		11.99	
Right angular gyrus	54	-69	27		11.46	
Left middle occipital gyrus	-36	-87	27		10.30	
Left middle cingulate cortex	-3	-39	36		11.43	
Right middle cingulate cortex	12	-39	36		9.92	
Left superior occipital gyrus	-21	-87	36		11.12	
Right cuneus	12	-87	39		9.93	
Right fusiform gyrus	30	-42	-12		9.55	
Right inferior temporal cortex	42	-69	-9		9.37	
Left Precuneus	-9	-57	15		9.17	
Cluster 2				48		< .001 FWE
Left inferior orbital frontal gyrus	-30	33	-18		9.78	
Cluster 3				53		< .001 FWE
Right hippocampus	24	-9	-18		9.01	
Cluster 4				199		< .001 FWE
Left fusiform gyrus	-33	-39	-18		8.88	
Left hippocampus	-24	-9	-18		7.19	
Cluster 5				836		< .001 FWE
Right superior medial frontal gyrus	6	66	6		7.22	
Right medial orbital frontal gyrus	9	48	-6		6.51	
Left Superior medial frontal gyrus	-3	63	24		7.74	
Left superior frontal gyrus	-15	57	33		7.46	
Left medial orbital frontal gyrus	-3	36	-12		7.15	
Left anterior cingulate cortex	0	39	-3		6.60	
Left gyrus rectus	-6	24	-18		7.04	
Right middle frontal gyrus	30	33	45		6.78	
Right superior frontal gyrus	15	57	36		5.86	
Right gyrus rectus	6	24	-21		6.39	
Cluster 6				178		< .001 FWE

Right middle temporal gyrus	54	-6	-18		7.80	
Right middle temporal pole	45	12	-33		7.79	
Right inferior temporal gyrus	51	0	-33		6.84	
Cluster 7				115		$< .001_{FWE}$
Left middle temporal pole	-45	12	-33		7.53	
Left middle temporal gyrus	-60	-9	-18		7.51	
Cluster 8				18		$< .001_{FWE}$
Left insular cortex	-36	-18	15		7.25	
Cluster 9				12		$.001_{FWE}$
Right inferior orbital frontal gyrus	33	36	-15		6.99	
Cluster 10				12		$.001_{FWE}$
Left middle frontal gyrus	-36	15	51		6.31	

Labeling clusters (the most significant local maxima for each area) obtained from activation maps thresholded at $p < 0.05$ FWE-corrected using Automated Anatomical Labeling (AAL) [17]. [N] – region of interest as listed in Table 3. p_{FWE} – cluster-level FWE-corrected over the entire brain volume.

REFERENCES

1. Zink, C. F., Pagnoni, G., Martin, M. E., Dhamala, M. & Berns, G. S. Human striatal response to salient nonrewarding stimuli. *J. Neurosci.* **23**, 8092–8097 (2003).

2. Münte, T. F. *et al.* Nucleus accumbens is involved in human action monitoring: evidence from invasive electrophysiological recordings. *Front. Hum. Neurosci.* **1:1**, doi: 10.3389/neuro.09.011.2007 (2008).

3. Downar, J., Mikulis, D. J. & Davis, K. D. Neural correlates of the prolonged salience of painful stimulation. *Neuroimage* **20**, 1540–1551 (2003).

4. Garrison, J., Erdeniz, B. & Done, J. Prediction error in reinforcement learning: A meta-analysis of neuroimaging studies. *Neurosci. Biobehav. Rev.* **37**, 1297–1310 (2013).

5. Bartra, O., McGuire, J. T. & Kable, J. W. The valuation system: A coordinate-based meta-analysis of BOLD fMRI experiments examining neural correlates of subjective value. *Neuroimage* **76**, 412–427 (2013).

6. Fouragnan, E., Retzler, C. & Philiastides, M. G. Separate neural representations of prediction error valence and surprise: Evidence from an fMRI meta-analysis. *Hum. Brain Mapp.* **39**, 2887–2906 (2018).

7. Wilson, R. P. *et al.* The neural substrate of reward anticipation in health: A meta-analysis of fMRI findings in the monetary incentive delay task. *Neuropsychol. Rev.* **28**, 496–506 (2018).

8. Syed, E. C. J. *et al.* Action initiation shapes mesolimbic dopamine encoding of future rewards. *Nat. Neurosci.* **19**, 34–36 (2015).

9. Mink, J. W. The basal ganglia: focused selection and inhibition of competing motor programs. *Prog Neurobiol* **50**, 381–425 (1996).

10. Alexander, G. E., DeLong, M. R. & Strick, P. L. Parallel organization of functionally segregated circuits linking basal ganglia and cortex. *Annu Rev Neurosci* **9**, 357–381 (1986).

11. Schultz, W., Dayan, P. & Montague, P. R. A neural substrate of prediction and reward. *Science (80-.).* **275**, 1593–1599 (1997).

12. Albin, R. L., Young, A. B., Penney, J. B., Roger, L. A. & Young, B. B. The functional anatomy of basal ganglia disorders. *Trends Neurosci* **12**, 366–375 (1989).

13. Haber, S. N. The place of dopamine in the cortico-basal ganglia circuit. *Neuroscience* **282**, 248–257 (2014).

14. Björklund, A. & Dunnett, S. B. Dopamine neuron systems in the brain: an update. *Trends Neurosci.* **30**, 194–202 (2007).

15. Parker, N. F. *et al.* Reward and choice encoding in terminals of midbrain dopamine neurons depends on striatal target. *Nat. Neurosci.* **19**, 845–854 (2016).

16. Coddington, L. T. & Dudman, J. T. The timing of action determines reward prediction signals in identified midbrain dopamine neurons. *Nat. Neurosci.* **21**, 1563–1573 (2018).

17. Tzourio-Mazoyer, N. *et al.* Automated anatomical labeling of activations in SPM using a macroscopic anatomical parcellation of the MNI MRI single-subject brain. *Neuroimage* **15**, 273–289 (2002).
