# Supplementary material for: Movement errors during skilled motor performance engage distinct prediction error mechanisms
Source: Commun Biol. 2020 Dec 11;3:763. doi: 10.1038/s42003-020-01465-4 (PMC7732826; doi:10.1038/s42003-020-01465-4)
Supplement: Supplementary file 2 — Reporting Summary [file 42003_2020_1465_MOESM2_ESM.pdf]

nature research

Corresponding author(s): Ella Gabitov & Julien Doyon

Last updated by author(s): Oct 19, 2020

Reporting Summary

Nature Research wishes to improve the reproducibility of the work that we publish. This form provides structure for consistency and transparency in reporting. For further information on Nature Research policies, see Authors & Referees and the Editorial Policy Checklist .

Statistics

For all statistical analyses, confirm that the following items are present in the figure legend, table legend, main text, or Methods section.

n/a	Confirmed	
☐	☒	The exact sample size (*n*) for each experimental group/condition, given as a discrete number and unit of measurement
☐	☒	A statement on whether measurements were taken from distinct samples or whether the same sample was measured repeatedly
☐	☒	The statistical test(s) used AND whether they are one- or two-sided<br>*Only common tests should be described solely by name; describe more complex techniques in the Methods section.*
☐	☒	A description of all covariates tested
☐	☒	A description of any assumptions or corrections, such as tests of normality and adjustment for multiple comparisons
☐	☒	A full description of the statistical parameters including central tendency (e.g. means) or other basic estimates (e.g. regression coefficient) AND variation (e.g. standard deviation) or associated estimates of uncertainty (e.g. confidence intervals)
☐	☒	For null hypothesis testing, the test statistic (e.g. *F*, *t*, *r*) with confidence intervals, effect sizes, degrees of freedom and *P* value noted<br>*Give P values as exact values whenever suitable.*
☒	☐	For Bayesian analysis, information on the choice of priors and Markov chain Monte Carlo settings
☒	☐	For hierarchical and complex designs, identification of the appropriate level for tests and full reporting of outcomes
☒	☐	Estimates of effect sizes (e.g. Cohen's *d*, Pearson's *r*), indicating how they were calculated

Our web collection on statistics for biologists contains articles on many of the points above.

Software and code

Policy information about availability of computer code

Data collection	MATLAB R2014A, https://www.mathworks.com;<br>Cogent, http://www.vislab.ucl.ac.uk/cogent.php.
Data analysis	SPM12, http://www.fil.ion.ucl.ac.uk/spm/software/spm12;<br>MarsBar toolbox for SPM, http://marsbar.sourceforge.net;<br>Functional Imaging Visualization Environment toolbox for SPM (FIVE), http://mrtools.mgh.harvard.edu.

For manuscripts utilizing custom algorithms or software that are central to the research but not yet described in published literature, software must be made available to editors/reviewers. We strongly encourage code deposition in a community repository (e.g. GitHub). See the Nature Research guidelines for submitting code & software for further information.

Data

Policy information about availability of data

All manuscripts must include a data availability statement. This statement should provide the following information, where applicable:
- Accession codes, unique identifiers, or web links for publicly available datasets
- A list of figures that have associated raw data
- A description of any restrictions on data availability

All data and computer code are available upon reasonable request from the corresponding authors. Requests should be addressed to E.G. (gabitovella@gmail.com) or J.D. (julien.doyon@mcgill.ca).

Field-specific reporting

Please select the one below that is the best fit for your research. If you are not sure, read the appropriate sections before making your selection.

- [x] Life sciences
- [ ] Behavioural & social sciences
- [ ] Ecological, evolutionary & environmental sciences

For a reference copy of the document with all sections, see nature.com/documents/nr-reporting-summary-flat.pdf

Life sciences study design

All studies must disclose on these points even when the disclosure is negative.

Sample size	The current report is based on the analyses of data collected during the training session from an fMRI experiment published elsewhere (Albouy et al., 2015). The sample included fifty-five healthy young adults (mean age: 24.1 ± 3.5 years, 34 females).
Data exclusions	Data from one participant was excluded due to excessive head movement. From the fifty-four participants whose data were analyzed in the current study, 52 of them completed at least one performance block (out of 14) without any error (mean = 7.02 errorless blocks, s.e.m. = 0.40). Errors that met the criteria for analyses were detected within the data of 49 participants (mean = 4.14 errors, s.e.m. = 0.30).
Replication	To estimate the reliability of our findings, we analyzed data of a sub-group of participants (N = 28) who were not only trained but also retested on the same motor sequence two hours later. The analysis of the training session allowed us to determine to what degree our results are reproducible in a smaller sample, whereas data acquired separately during the retest session were used to conduct a replication study. The pattern of the results derived from these analyses was similar to that of our main study.
Randomization	Periods without errors that were used as a control condition were determined by applying pseudo-random matching procedure. An additional set of such periods was generated to test the specificity of the observed changes to errors.
Blinding	N/A

Reporting for specific materials, systems and methods

We require information from authors about some types of materials, experimental systems and methods used in many studies. Here, indicate whether each material, system or method listed is relevant to your study. If you are not sure if a list item applies to your research, read the appropriate section before selecting a response.

Materials & experimental systems

n/a	Involved in the study	
x		Antibodies
x		Eukaryotic cell lines
x		Palaeontology
x		Animals and other organisms
	x	Human research participants
x		Clinical data

Methods

n/a	Involved in the study	
x		ChIP-seq
x		Flow cytometry
	x	MRI-based neuroimaging

Human research participants

Policy information about studies involving human research participants

Population characteristics	Fifty-five healthy young (mean age: 24.1 ± 3.5 years, 34 females) right-handed (Oldfield, 1971) volunteers were recruited to participate in the study. Participants reported no history of medical, neurological or psychiatric disease. None of the participants were taking medications at the time of testing. Also, none received formal training on a musical instrument or as a typist. All participants had normal quality of sleep, as assessed by the Pittsburgh Sleep Quality Index questionnaire (Buysse et al., 1989) and the St. Mary Hospital questionnaire (Ellis et al., 1981).
Recruitment	Participants for the study were recruited by local advertisements without limiting gender, ethnicity, level of education (except musicians), etc. Yet, our sample included more females than males, which is consistent with the observation that, generally, volunteers tend to be females. Since we were interested in exploring fundamental neurophysiological mechanisms of prediction error signals and their contribution to error processing in humans, we did not test for potential sex differences. Given the target population (healthy young adults between 18 and 35 years old) and the location of the study (Research center of the University of Montreal), most of the participants were university students and all of them lived in Montreal or its suburbs. The task used in the study did not require special abilities except for being able to type on a keyboard. Such ability is ubiquitous in modern society and is not an exclusive characteristic of university students. To control for proficiency levels in typing skills, volunteers who received formal training on a musical instrument or were typists (i.e., by default used all five fingers of both hands when typing) were screened out. Since the study was conducted using functional magnetic resonance imaging, participants also should have been comfortable with performing the task when lying in the scanner.
Ethics oversight	All participants gave their written informed consent to take part in the study, which was approved by the Research ethics board of the RNQ (Regroupement Neuroimagerie Québec). All procedures were in accordance with the approved guidelines and regulations.

Note that full information on the approval of the study protocol must also be provided in the manuscript.

Magnetic resonance imaging

Experimental design

Design type	Participants were scanned, using functional magnetic resonance imaging, when they were engaged in the motor sequence task (block design).
Design specifications	14 successive blocks of task performance comprising 60 key-presses, i.e., equivalent to 12 repetitions of the correctly performed and competed sequence, separated by 15-second periods of rest.
Behavioral performance measures	Performance was registered by saving the code-number (i.e., 1, 2, 3 or 4) and time of each key press. Detailed descriptive statistics, including group mean and standard deviation, were carried out for the number of correct sequences, number of errors, number of keys within the errors and intervals between key presses.

Acquisition

Imaging type(s)	Structural and functional magnetic resonance imaging
Field strength	3T
Sequence & imaging parameters	Functional MRI-series were acquired using a 3.0 T TIMTRIO scanner system (Siemens, Erlangen, Germany), equipped with a 32-channel head coil. T2*-weighted axial fMRI images were obtained with a gradient echo-planar sequence using interleaved acquisition mode in ascending direction (TR = 2650 ms, TE = 30 ms, FA = 90°, FoV = 220×220 mm2, matrix size = 64×64×43, voxel size = 3.4×3.4×3 mm3, 10% inter-slice gap). T1-weighted sagittal 3D MP-RAGE structural images were also obtained (TR = 2300 ms, TE = 2.98 ms, TI = 900 ms, FA = 9°, FoV = 256×256 mm2, matrix size = 256× 256×176, voxel size = 1×1×1 mm3).
Area of acquisition	Whole-brain scan

Diffusion MRI ☐ Used ☒ Not used

Preprocessing

Preprocessing software	The structural and functional images were converted to Neuroimaging Informatics Technology Initiative (NIfTI) format using MRIcron (University of South Carolina). Preprocessing and statistical analysis of the data were carried out with SPM12 (http://www.fil.ion.ucl.ac.uk/spm/software/spm12/; Wellcome Trust Centre for Neuroimaging, London, UK) operating under Matlab R2014a (The Mathworks, Inc., Natick, MA).
Normalization	Following segmentation and skull-stripping of the structural data, functional images were coregistered to the individual skull-stripped 3-D anatomical image and normalized to the Montreal Neurological Institute (MNI) space using parameters obtained from the segmentation procedure.
Normalization template	For normalization procedure, we used ICBM152 - a default template in SPM12

Noise and artifact removal

During initial preprocessing, functional volumes were realigned using a least squares approach and a six parameter (rigid body) spatial transformation to correct for movement-related variance. Movement parameters derived from realignment procedure were included as covariates of no interest in individual (1st level) statistical models. A high-pass filter of 128 seconds was used to remove low-frequency noise.

Volume censoring

Volume censoring was applied using the Artifact Detection Tools with normalized z-threshold of 5 and movement threshold of 0.9 mm.

Statistical modeling & inference

Model type and settings

Statistical analyses of fMRI time-series consisted in a two-stage summary statistics model (Holmes and 564 Friston, 1998).

In the first stage, BOLD signal changes were estimated for each subject independently using a fixed effect general linear modeling (GLM). We used a mixed block/event-related design (Visscher et al., 2003) to separate transient activity related to trials of interest (errors/ sequences) from sustained task-related activity during continuous motor sequence practice. Each model comprised covariate for performance periods represented as a boxcar function, time-locked to the onset and duration of each block, and covariates for trials represented as a stick function, i.e., zero duration, time locked to the trial onset. To minimize the effect of block onset transients (Fox et al., 2005), additional covariate represented as a stick function time-locked to task onset ("GO" cue) was included in the models. All covariates were convolved with a hemodynamic impulse response function (HRF). Serial correlations in fMRI signal were estimated through a restricted maximum likelihood (ReML) algorithm using a first order autoregressive plus white noise model.

In the second stage, t-maps from the 1st level models were carried forward to the random effects analyses to assess the consistency of effects between subjects (group-level analyses).

Effect(s) tested

The inferences about sustained task-related activity and changes time-locked to errors were done using a one-sample t test. To get insight into temporal characteristics of error-related network, comparisons between activity changes immediately before, during and after errors were performed using a one-way within-subject ANOVA.

Specify type of analysis: ☐ Whole brain ☐ ROI-based ☒ Both

Anatomical location(s)

Regions of interest (ROIs) were defined within the brain areas involved in motor sequence production (Doyon et al., 2018), error processing (Dosenbach et al., 2006; Neta et al., 2015) and inhibitory control (Aron and Poldrack, 2006) based on activation maps of task- and error-specific activity (errorless performance versus rest and errors versus rest/matching sequences, respectively). In that way, the ROIs within these networks were localized independently of the whole-brain analyses of changes before and after error onset. Additional areas were identified based on activation maps of significant changes during the post-error performance recovery (errors versus first post-error correctly performed and competed sequences).

Statistic type for inference
(See Eklund et al. 2016)

Unless otherwise stated, activation maps were thresholded at $p \leq 0.001$. Statistical inferences were performed at the cluster level using p values family-wise error rate (FWE)-corrected for multiple comparisons over the entire brain.

Correction

Family-wise error rate (FWE)-correction for multiple comparisons over the entire brain.

Models & analysis

n/a	Involved in the study
☒	☐ Functional and/or effective connectivity
☒	☐ Graph analysis
☒	☐ Multivariate modeling or predictive analysis
